# Association of non-malignant diseases with thrombocytosis: a prospective cohort study in general practice

**DOI:** 10.3399/bjgp20X713501

**Published:** 2020-11-17

**Authors:** Cansu Clarke, Willie Hamilton, Sarah Price, Sarah ER Bailey

**Affiliations:** University of Exeter Collaboration for Academic Primary Care and the South West Peninsula Deanery, College of Medicine and Health, University of Exeter, Exeter.; DISCO Cancer Diagnostics Group, University of Exeter Medical School, College House, St Luke’s Campus, University of Exeter, Exeter.; DISCO Cancer Diagnostics Group, University of Exeter Medical School, College House, St Luke’s Campus, University of Exeter, Exeter.; DISCO Cancer Diagnostics Group, University of Exeter Medical School, College House, St Luke’s Campus, University of Exeter, Exeter.

**Keywords:** cohort studies, diagnosis, incidence, platelet count, prevalence, primary care, thrombocytosis

## Abstract

**Background:**

Thrombocytosis is an excess of platelets, which is diagnosed as a platelet count >400 × 10^9^/l. An association of thrombocytosis with undiagnosed cancer has recently been established, but the association with non-malignant disease has not been studied in primary care.

**Aim:**

To examine, in English primary care, the 1-year incidence of non-malignant diseases in patients with new thrombocytosis and the incidence of pre-existing non-malignant diseases in patients who develop new thrombocytosis.

**Design and setting:**

Prospective cohort study using English Clinical Practice Research Datalink data from 2000 to 2013.

**Method:**

Newly incident and pre-existing rates of non-malignant diseases associated with thrombocytosis were compared between patients with thrombocytosis and age- and sex-matched patients with a normal platelet count. Fifteen candidate non-malignant diseases were identified from literature searches.

**Results:**

In the thrombocytosis cohort of 39 850 patients, 4579 (11.5%) were newly diagnosed with any one of the candidate diseases, compared with 443 out of 9684 patients (4.6%) in the normal platelet count cohort (relative risk [RR] 2.5, 95% confidence intervals [CI] = 2.3 to 2.8); iron-deficiency anaemia was the most common new diagnosis (4.5% of patients with thrombocytosis, RR 4.9, 95% CI = 4.0 to 6.1). A total of 22 612 (57.0%) patients with thrombocytosis had a pre-existing non-malignant diagnosis compared with 4846 patients (50%) in the normal platelet count cohort (odds ratio 1.3, 95% CI = 1.2 to 1.4). There was no statistically significant difference in cancer diagnoses between patients with and without pre-existing disease in the thrombocytosis cohort.

**Conclusion:**

Thrombocytosis is associated with several non-malignant diseases. Clinicians can use these findings as part of their holistic diagnostic approach to help guide further investigations and management of patients with thrombocytosis.

## INTRODUCTION

Thrombocytosis is a higher than normal value of platelets in human plasma.^1^ The currently accepted normal range in the UK is 150–400 × 10^9^/l.^1^ Platelets are acute-phase reactants; they may increase in response to infection, inflammation, bleeding, and tumours.^2^^–^^5^ Their main roles are haemostasis and clot formation, and they have a possible additional role in immune response.^4^^,^^6^ The association of thrombocytosis with cancer has been recognised, with 11% of patients with thrombocytosis identified in primary care being diagnosed with cancer in the following year.^7^ It is less well understood which other diagnoses are present in the 89% of patients with thrombocytosis who do not have cancer. Secondary care studies suggest links between thrombocytosis and thrombotic conditions such as stroke,^8^ autoimmune diseases such as coeliac disease,^9^^,^^10^ vasculitic conditions such as giant cell arteritis,^11^ and inflammatory diseases such as rheumatoid arthritis.^12^ However, these associations are based on little published evidence. It would be clinically helpful to know the alternative diagnoses associated with thrombocytosis to guide clinical decision making in primary care, particularly to avoid investigation for possible cancer when the patient has an existing condition potentially explaining the thrombocytosis. No previous studies in primary care have explored the association of thrombocytosis with non-malignant diseases.

The aims of this study were to examine, in patients with new thrombocytosis and those with a normal platelet count in primary care, the 1-year incidence of non-malignant diseases and the incidence of pre-existing non-malignant diseases.

## METHOD

### Selection and description of participants

This prospective cohort study used a random data sample from the UK Clinical Practice Research Datalink (CPRD), consisting of anonymised electronic records of patients’ clinical details taken from English general practices.^13^^,^^14^ This was a secondary analysis of a CPRD dataset used in a study that investigated the risk of undiagnosed cancer in patients with thrombocytosis.^7^ In that study, 40 000 patients with thrombocytosis, defined as a platelet count of 400–999 × 10^9^/l, and recorded from 2000 to 2013, were randomly selected from the CPRD. Also, a further 10 000 patients with a normal platelet count, matched for sex, year of birth, and general practice to a random quarter of the 40 000 patients with thrombocytosis, were selected as a comparison cohort. Therefore, the cohort included 50 000 patients who had had a full blood count and no previous thrombocytosis. The index date was the date at which a patient first had thrombocytosis. The index date for the comparator cohort was the date of their normal platelet count (150–400 × 10^9^/l) closest in time to the index date of their matched case in the thrombocytosis cohort. Exclusions were patient age <40 years, low platelet count values (<150 × 10^9^/l), and clinically improbable platelet counts (>1000 × 10^9^/l). These exclusions were applied to match the sample used for the original study and therefore juxtapose the results against those of the original study. Patients aged <40 years were excluded from the sample in the original study, which accurately speculated that cancer is rare below the age of 40 and tends to be familial in this group.

**Table:** How this fits in

Thrombocytosis has recently emerged as a risk marker of undiagnosed cancer in patients in primary care. However, 89% of patients with thrombocytosis do not have undiagnosed cancer. This study estimates the link between thrombocytosis and non-malignant diseases. Primary care clinicians can use the results of this study as a clinical aid to look for and to diagnose associated diseases other than cancer in patients with thrombocytosis.

### Outcome variables

The primary outcome was diagnosis of any of 15 non-malignant diseases in the patient records at any time before or in the first year after the index date. The process of identification of candidate non-malignant diseases began with a literature search of diseases associated with thrombocytosis. The search inclusion criteria were high platelet count, non-malignant diseases, adults, the year 2000 onwards, and studies published in English from Europe, the USA, and Australia, chosen because they have healthcare systems comparable with that of the UK.

The 15 candidate diseases were ischaemic heart disease, rheumatoid arthritis, chronic obstructive pulmonary disease (COPD), segmental colitis associated with diverticulosis, inflammatory bowel disease, iron-deficiency anaemia, Raynaud’s phenomenon, giant cell arteritis, thromboembolic disease, coeliac disease, sarcoidosis, granulomatosis with polyangiitis, chronic hepatitis B, functional hyposplenism, and ankylosing spondylitis. Accurate CPRD codelists for each disease were established using validated methods.^15^ CPRD records from 2000 to 2013 were searched using the 15 disease codelists.

### Statistical methods

Simple descriptive statistics were used to summarise age and sex for the thrombocytosis and normal-platelet cohorts, presenting the median and interquartile range. The diagnosis date for each candidate disease was taken as the first record in time for that disease. Diseases first coded before the index date were considered pre-existing diseases; those coded for the first time in the first year after the index date were considered incident disease. Differences between the thrombocytosis and normal-platelet cohorts were explored, in terms of prevalence and 1-year incidence of each disease. The results are reported as odds ratios (OR) and risk ratios (RR), respectively, estimated from frequency tables. For rare candidate diseases with fewer than five cases, differences between thrombocytosis and normal-platelet groups were examined using Fisher’s 2-sided exact test (*P*<0.05). The proportion of patients with and without pre-existing disease who were later diagnosed with cancer was compared with a χ^2^ test. All analyses were performed using Stata (version 14). The RECORD statement was used as a reporting guideline.^16^

## RESULTS

**Figure 1.**
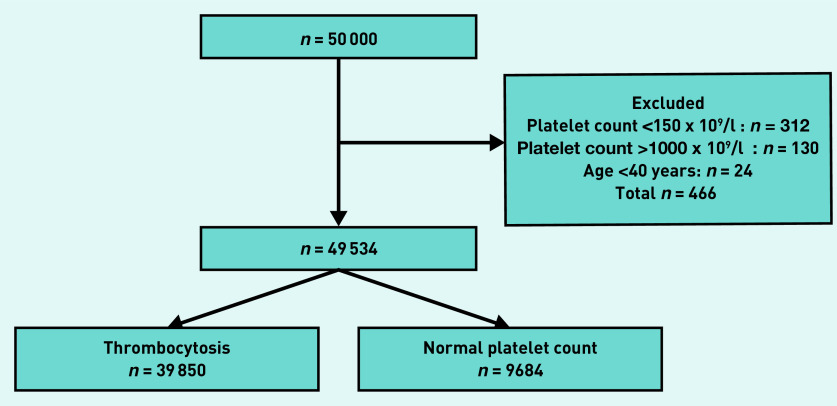
***The number of patients included in the cohorts and excluded for having a platelet count out of eligible range and/or being <40 years old.***

There were initially 50 000 eligible patients, 466 of whom were excluded: 24 were <40 years, 312 had a low platelet count (<150 × 10^9^/l) and 130 had a clinically improbable platelet count (>1000 × 10^9^/l). Of the remaining 49 534 patients, 39 850 (80.4%) had thrombocytosis and 9684 (19.5%) had a normal platelet count (Figure 1). Median age and the proportion of male patients was comparable between the two groups (Table 1).

**Table 1.** Age and sex of patients in the thrombocytosis and normal platelet count cohorts, and proportion of patients in each cohort diagnosed with any one of the candidate diseases from 2000 to 2013

**Cohort**	**Total number *n* (%)**	**Male sex *n* (%)**	**Age, years**			**Number of patients with:**	
			**40–64 (%)**	**≥65 (%)**	**Median (IQR)**	**Pre-existing disease, *n* (%)**	**No pre-existing disease, *n* (%)**
**TH**	39 850 (80.4%)	12 709 (31.9%)	15 455 (38.8%)	24 395 (61.2%)	70 (59–79)	22 612 (56.7%)	17 238 (43.3%)
**NPC**	9684 (19.5%)	3032 (31.3%)	3701 (38.2%)	5983 (61.7%)	70 (59–80)	4846 (50.0%)	4838 (50.0%)

IQR = interquartile range. NPC = normal platelet count. TH = thrombocytosis.

### Prevalent disease

The occurrence of one or more of the candidate diseases associated with elevated platelet count was more common in the thrombocytosis cohort (22 612/39 850; 56.7%) than in the normal platelet count cohort (4846/9684; 50.0%; OR 1.3, 95% CI = 1.2 to 1.4), as were multiple diagnoses (Table 2). Inflammatory bowel disease, iron-deficiency anaemia, rheumatoid arthritis, COPD, and giant cell arteritis were more likely in patients with thrombocytosis than in those with a normal platelet count. In the thrombocytosis cohort, 190 (5.7%) patients with pre-existing disease and 2388 (6.5%) patients with no pre-existing disease were subsequently diagnosed with cancer; this difference was not statistically significant (*P* = 0.061), (data not shown).

**Table 2.** Number of patients who had one or more disease diagnoses in the thrombocytosis and normal platelet count cohorts

**Number of diseases diagnosed**	**Patients in cohort**		**Total**
	**TH (*N*= 39 850) *n* (%)**	**NPC (*N*= 9684) *n* (%)**	**(*N*= 49 534) *n* (%)**
1	31 031 (77.9)	8177 (84.4)	39 208 (79.2)
2	6293 (15.8)	1182 (12.2)	7475 (15.1)
3	1983 (5.0)	281 (2.9)	2264 (4.6)
4	464 (1.7)	39 (0.4)	503 (1.0)
5	72 (0.2)	5 (0.05)	77 (0.2)
6	7 (0.02)	0	7 (0.01)

NPC = normal platelet count. TH = thrombocytosis.

### Incidence of disease at 1-year

The 1-year incidence of each disease (those recorded for the first time in the year following new thrombocytosis) is shown in Table 3. The diseases with a statistically significantly greater risk of 1-year incidence in the thrombocytosis cohort compared with the normal platelet count cohort were: iron-deficiency anaemia 4.5% (RR 4.9, 95% confidence interval [CI] = 4.0 to 6.1); giant cell arteritis 2.2% (RR 5.0, 95% CI = 3.7 to 6.8); rheumatoid arthritis 0.9% (RR 5.0, 95% CI = 3.1 to 8.0); coeliac disease 0.2% versus 0.04% (*P*<0.001); inflammatory bowel disease 1.0% (RR 2.1, 95% CI = 1.6 to 2.9); and segmental colitis associated with diverticulosis 0.3% (RR 1.8, 95% CI = 1.1 to 3.0).

**Table 3.** One-year incidence and prevalence rates of disease for thrombocytosis and normal platelet count cohorts

**Disease**	**1-year incidence of disease**			**Pre-existing disease**		
	**TH (*N*= 39 850) *n* (%)**	**NPC (*N*= 9684) *n* (%)**	**RR (95% CI)**	**TH (*N*= 39 850) *n* (%)**	**NPC (*N*= 9684) *n* (%)**	**OR (95% CI)**
Thromboembolic disease	589 (1.5)	114 (1.2)	1.3 (1.0 to 1.5) *P* = 0.02	8943 (22.4)	2325 (24.0)	0.9 (0.9 to 1.0) *P* = 0.001
Ischaemic heart disease	524 (1.3)	113 (1.2)	1.1 (0.9 to 1.4) *P* = 0.24	4816 (12.1)	1144 (11.8)	1.0 (1.0 to 1.1) *P* = 0.46
Inflammatory bowel disease	386 (1.0)	44 (0.5)	2.1 (1.6 to 2.9) *P*<0.001	2232 (5.6)	406 (4.2)	1.4 (1.2 to 1.5) *P*<0.001
Iron-deficiency anaemia	1811 (4.5)	89 (0.9)	4.9 (4.0 to 6.1) *P*<0.001	2055 (5.2)	346 (3.6)	1.5 (1.3 to 1.7) *P*<0.001
Rheumatoid arthritis	370 (0.9)	18 (0.2)	5.0 (3.1 to 8.0) *P*<0.001	1479 (3.7)	155 (1.6)	2.4 (2.0 to 2.8) *P*<0.001
Giant cell arteritis	866 (2.2)	42 (0.4)	5.0 (3.7 to 6.8) *P*<0.001	1253 (3.1)	194 (2)	1.6 (1.4 to 1.9) *P*<0.0001
Segmental colitis associated with diverticulosis	136 (0.3)	18 (0.2)	1.8 (1.1 to 3.0) *P* = 0.01	950 (2.4)	202 (2.0)	1.1 (1.0 to 1.3) *P* = 0.08
Chronic obstructive pulmonary disease	77 (0.2)	11 (0.1)	1.7 (0.9 to 3.2) *P* = 0.09	488 (1.2)	62 (0.6)	1.9 (1.5 to 2.6) *P*<0.001
Raynaud’s syndrome	39 (0.1)	4 (0.04)	*P* = 0.12	385 (1.0)	83 (0.9)	*P* = 0.35
Coeliac disease	75 (0.2)	4 (0.04)	*P*<0.001	107 (0.3)	34 (0.4)	*P* = 0.17
Other^a^	34 (0.09)	6 (0.06)	—	211 (0.5)	43 (0.4)	—
At least one of the above^b^	4579 (11.5)	443 (4.6)	2.5 (2.3 to 2.8) *P*<0.001	22 612 (56.7)	4846 (50.0)	1.3 (1.2 to 1.4) *P*<0.001

a Other includes ankylosing spondylitis, chronic hepatitis B, sarcoidosis, and granulomatosis with polyangiitis.

b The number of patients with at least one disease diagnosed. NPC = normal platelet count. OR = odds ratio. RR = risk ratio. TH = thrombocytosis. — = no statistical methods applied here due to the heterogeneity of the ‘other’ group.

The risk of having any one of the diseases in the first year after index date is 2.5 times greater in patients with thrombocytosis compared with those with a normal platelet count (RR 2.5, 95% CI = 2.3 to 2.8).

## DISCUSSION

### Summary

This primary care cohort study has examined the occurrence of non-malignant diseases in patients with thrombocytosis and those with a normal platelet count in primary care to inform clinical decision making when investigating an unexpected thrombocytosis. A total of 56.7% of those with thrombocytosis had a pre-existing condition, such as inflammatory bowel disease, which may explain the raised platelets. However, that figure needs to be tempered by the finding that 50.0% of patients without thrombocytosis also had one of the conditions linked with raised platelets. Furthermore, there was no statistically significant difference in the proportion of patients with or without pre-existing disease who were diagnosed with cancer.

In terms of incident diseases, the thrombocytosis cohort was more than twice as likely to have a first record of one of the candidate diseases in the year after newly recorded thrombocytosis, with iron-deficiency anaemia being the most common condition. The results presented in this study can guide clinicians on when not to investigate for suspected cancer, if there is a reasonable alternative explanation for the thrombocytosis. They suggest that cancer should be considered with a raised platelet count, even with pre-existing conditions, though the decision whether to investigate for possible cancer will depend on how active the pre-existing disease is, as well as whether there are other symptoms suggestive of cancer. The results also provide guidance on other conditions to consider, if cancer has been ruled out.

### Strengths and limitations

The CPRD is a well-established high-quality data source. A strength of this study is its size, providing the opportunity to examine rarer diseases.^14^ These findings are also generalisable to the adult UK primary care population because the patients in the sample are representative of the population to which the results can be applied. The candidate diseases were assembled using published literature from secondary care. Though the search was systematic, it remains possible that other diseases associated with thrombocytosis were omitted. Similarly, knowing of the association between candidate diseases and thrombocytosis does not mean all patients with a record of one of the diseases actually had their thrombocytosis caused by the disease. Indeed, the similarity between prevalence of candidate diseases in the thrombocytosis and the comparison groups strongly suggests many of the conditions found were not associated with the rise in platelets.

CPRD data are observational, meaning there may be elements of observer bias or measurement errors. The risk of this was considerably reduced because blood test results are electronically submitted into patient records; however, this research was reliant on accurate recording of the candidate diseases, with the possibility of some missing data. It is therefore likely that the incidence and prevalence figures from this study are underestimates, though it is very unlikely that the differences between the thrombocytosis and normal platelet results can be explained by differential under-recording. All patients in this study had had a full blood count. The reasons for ordering their blood tests are not known. Patients who have had an investigative blood test — even one as ubiquitous as a full blood count — are on average more ill than those that have not had one,^7^ which could have generated selection bias. To mitigate this, the comparison group comprised patients who had also had a full blood count.

Another limitation is the assumption that identified prevalent diseases were chronic, so that at the time of the diagnosis of thrombocytosis, patients still had the disease. A greater proportion of the sample were female; 68.1% in the thrombocytosis cohort, and 69.0% in the normal platelet count cohort. Females are more likely to use primary care, and to have a blood test.^17^

Furthermore, females have a higher baseline platelet count and therefore what is recorded as thrombocytosis in some females could be considered ‘normal’.^18^

### Comparison with existing literature

This study addresses the clinical problem arising from the authors’ previous findings that thrombocytosis is an important risk marker for cancer.^7^ That finding prompted clinical uncertainty about which patients with thrombocytosis should be investigated, and how this could be done. This aim of the present study, which was the first step in answering this uncertainty, was to identify which non-malignant diseases are associated with thrombocytosis in primary care.

No studies were found that reported thrombocytosis with diseases specifically in a primary care setting; in secondary care, thrombocytosis has been found to be a cardiovascular risk factor,^19^ and linked to rheumatoid arthritis.^20^ It is a biomarker for severe COPD, though the mechanism for this is uncertain.^21^

There is a commonly reported link between thrombocytosis, coagulopathy,^8^^,^^22^^,^^23^ and inflammation in conditions such as arthritis,^20^ giant cell arteritis,^11^ thrombotic disease,^24^ diverticulitis,^25^ coeliac disease,^9^ inflammatory bowel disease,^23^ and iron-deficiency anaemia.^8^^,^^26^

### Implications for practice

What should be done in primary care, when a patient has thrombocytosis, often as an unexpected result? The previously reported 11% risk of cancer will probably remain the first diagnostic consideration for clinicians, even after the findings of this study. However, more than half of patients will have a prevalent condition that may (or may not) explain the platelet findings. Fortunately, the conditions that are more frequent with thrombocytosis than with a normal platelet count generally appear in both the prevalent and incident lists. These conditions, like inflammatory bowel disease, giant cell arteritis, and rheumatoid arthritis, may be considered likely explanations for unexpected thrombocytosis. It seems reasonable to defer cancer investigation in a patient with one of those existing conditions, unless the patient has symptoms of a possible cancer.

If there are no plausible explanations for a patient’s thrombocytosis, the investigation strategy will probably initially focus on possible cancer, with its 11% risk. However, there may be clues from other parts of the full blood count or an accompanying abnormal inflammatory marker. Iron-deficiency anaemia may be the explanation for the thrombocytosis (newly identified in 4.5% of patients with thrombocytosis), though it is also a clear marker for possible colorectal cancer, and would usually be urgently investigated further. Similarly, a raised inflammatory marker may point to giant cell arteritis (newly identified in 2.2% of patients with thrombocytosis), though the small possibility of myeloma must be considered. Inflammatory bowel disease, coeliac disease, or segmental colitis with diverticulosis were newly diagnosed in 1.5% of patients with thrombocytosis; bowel investigation may therefore be warranted in those with suggestive symptoms.

However, none of these recommendations is absolute: GPs will want to use their clinical experience to supplement these findings in the individual patient. It needs also to be remembered that most patients with thrombocytosis will transpire to have none of these diseases.
